# Development and Characterisation of a Four-Plex Assay to Measure *Streptococcus pyogenes* Antigen-Specific IgG in Human Sera

**DOI:** 10.3390/mps5040055

**Published:** 2022-06-27

**Authors:** Alexander J. Keeley, Martina Carducci, Luisa Massai, Mariagrazia Pizza, Thushan I. de Silva, Danilo G. Moriel, Omar Rossi

**Affiliations:** 1 Department of Clinical Research, London School of Hygiene and Tropical Medicine, London WC1E 7HT, UK; alexander.keeley@lshtm.ac.uk; 2 Department of Infection, Immunity and Cardiovascular Disease, University of Sheffield, Sheffield S10 2TN, UK; t.desilva@sheffield.ac.uk; 3 Vaccines and Immunity Theme, Medical Research Unit the Gambia at the London School of Hygiene and Tropical Medicine, Atlantic Boulevard, Fajara, P. O. Box 273, The Gambia; 4 GSK Vaccines Institute for Global Health (GVGH), Via Fiorentina 1, 53100 Siena, Italy; martina.x.carducci@gsk.com (M.C.); luisa.x.massai@gsk.com (L.M.); mariagrazia.x.pizza@gsk.com (M.P.); danilo.x.moriel-gomes@gsk.com (D.G.M.)

**Keywords:** GAC, IgG measurement, Luminex, Slo, SpyCEP, *Streptococcus pyogenes*, strep A, SpyAD, vaccine

## Abstract

The measurement of antibodies to vaccine antigens is crucial for research towards a safe and effective vaccine for *Streptococcus pyogenes* (Strep A). We describe the establishment and detailed characterisation of a four-plex assay to measure IgG to the Strep A vaccine antigens SpyCEP, Slo, SpyAD and GAC using the Luminex multiplex platform. A standard curve was established and characterized to allow the quantification of antigen-specific IgG. Assay specificity, precision, linearity, reproducibility and repeatability were determined via the measurement of antigen-specific IgG from pooled human serum. The assay is highly specific, reproducible and performs well across a large range of antibody concentrations against all four antigens. It is, therefore, suitable for future clinical trials in humans with a four-component vaccine, as well as for seroepidemiological studies to gain insights into naturally occurring immunity.

## 1. Introduction

*Streptococcus pyogenes* (Strep A) is a pathogen responsible for half a million deaths annually [1,2]. The spectrum of clinical manifestation ranges from asymptomatic colonisation, through mild but highly prevalent pharyngeal and skin and soft tissue infection, to severe and frequently fatal invasive disease. Furthermore, pathological immune reaction to Strep A infection can cause glomerulonephritis and acute rheumatic fever, which may lead to the chronic condition of rheumatic heart disease (RHD). RHD has a devastating global impact and has been neglected by research funding [2,3]. The vast majority of morbidity and mortality from Strep A occurs in low- and middle-income countries [1,2]. However, disease caused by Strep A is theoretically vaccine preventable, and, therefore, a World Health Assembly resolution in 2018 declared that the development of a safe and effective vaccine against Strep A is a major global health priority [4]. A leading vaccine candidate is under development by the GSK Vaccines Institute for Global Health (GVGH), composed of three recombinant proteins, including Streptolysin O (Slo), *S. pyogenes* cell envelope protein (SpyCEP), *S. pyogenes* adhesion and division protein (SpyAD) and the group A carbohydrate (GAC) conjugated to the recombinant CRM protein [5,6]. All four antigens are present and highly conserved in a global analysis of Strep A genomes [7]. As part of the efforts to measure immunological end points from future vaccine trials, an assay to quantify IgG titres to Strep A vaccine antigens is required. Furthermore, reliable and reproducible serological investigations are needed for research towards understanding natural immunity, including from within longitudinal cohort studies, in order to identify serological correlates of protection [8,9]. Luminex technology, whereby antigens are coupled to magnetic beads with unique florescent properties allowing for multiplex serological assays to be performed in the liquid phase, has recently been used to measure antibodies to Strep A antigens with high specificity and precision and across a large dynamic range [10,11].

We herein describe the development and in-depth characterisation of an assay capable of measuring antibody responses elicited by human Strep A vaccines in clinical trials and in seroepidemiological studies using a multiplexed Luminex platform.

## 2. Materials and Methods

### 2.1. Antigens Preparation and Coupling

Four antigens were incorporated into the assay: Slo, SpyCEP, SpyAD and the group A carbohydrate (GAC). Antigens were purified and fully characterised analytically, as previously described [6,12,13]. A total of 10 µg of Slo and SpyCEP and 20 µg of SpyAD and Streptavidin (Thermo Fisher) were coupled to 1.25 × 10^6^ carboxylated MagPlex magnetic microspheres (Luminex Corporation, MC10012-30; -20; -12; 25, respectively, Table A1) following the Luminex carbodiimide protein coupling method [14]. Coupling concentrations were determined from previous optimisation experiments, evaluating different concentrations of each antigen and selecting the lowest still giving saturation of the signal. Using magnetic separation, the microspheres were washed with deionised water and resuspended in an activation buffer of 100 nM, pH 6.2 monophasic sodium phosphate (Sigma Darmstadt, Germany) and activated with an equal volume of 50 mg/mL N-hydroxysulfosuccinimide (Thermo Fisher Dartford, United Kingdom)) and 50 mg/mL 1-ethyl-3-(3-dimethylaminopropyl)carbodiimide hydrochloride (Thermo Fisher). Activated beads were washed twice with 50 mM pH 5.0 4-Morpholineethanesulfonic acid (MES), then incubated with the protein antigens or Streptavidin for 2 h at room temperature with rotational inversion. Coupled microspheres were then washed twice with PBS (phosphate buffered saline) with 0.05% tween (PBST, Calbiochem) and stored in PBST with 0.5% bovine serum albumin (Sigma) until use. The polysaccharide structure of GAC antigen means standard carbodiimide-mediated peptide coupling is not suitable. Therefore, following two washes in PBST, microspheres coupled to Streptavidin were coupled with biotinylated GAC. The biotinylated GAC and the Streptavidin-coupled microspheres were incubated for 1 h at room temperature with rotational inversion protected from light. The GAC coupled beads were then washed twice in PBST and resuspended in 1 mL of storage buffer and stored at 4 °C.

### 2.2. Quantification of Total Human IgG to Slo, SpyAD, SpyCEP and GAC

In setup experiments pooled human immunoglobulin (IVIG) preparations were tested to generate a working standard for the assay: two formulations of IVIG, Privigen (CSL Behring) and Octanorm (Octapharma) were tested at dilutions (2-fold apart, 23 dilution points) ranging from 1:100 (1 mg/mL) to 1:419,430,400 (0.24 ng/mL). In calibration experiments Privigen IVIG preparations were diluted 3-fold in PBS from 1:1000 (0.1 mg/mL), which was assigned an arbitrary value of 100 relative Luminex units/mL (RLU/mL), to 1:59,049,000. Varying primary and secondary incubation times (30, 45 and 60 min, respectively), and PE-labelled secondary antibody concentrations (1:50, 1:70, 1:100) were tested to select the optimal conditions, which have been used herein.

A 10-point, 3-fold serially diluted Privigen human IVIG working standard (starting from 1:1000 to 1:19,683,000) prepared in PBS was selected and used in all subsequent experiments. Test serum samples were prepared at 4 dilution points, 3-fold serial dilutions with lowest dilution of 1:100. A total of 50 mL of diluted standard or test sera were mixed with 10 mL of conjugated microsphere in PBST containing 2500 microspheres/region/well in a 96-well Greiner plate (Millipore Corporation, Watford United Kingdom) and incubated for 60 minutes at room temperature in the dark on a plate shaker at 750 rpm. Two blank wells containing PBS and microspheres only were included in each plate. After incubation, the microspheres were washed three times with 200 μL PBS. Each well was loaded with 50 μL of 2.5 μg/mL (1:70 dilution) R-phycoerythrin AffiniPure goat anti-human IgG, Fcγ fragment specific (Jackson Immunoresearch) in PBS and incubated for 30 minutes in the dark while shaking at 750 rpm. After incubation, the microspheres were washed three times with 200 μL PBS then resuspended in 100 μL PBS. Data were acquired in real time by Bioplex Manager 6.2 Software (Biorad, Watford, United Kingdom) using the Bioplex-200 reader. A 5PL parameter logistic curve was fit to the blank-subtracted median fluorescence intensity (MFI) values obtained for each standard curve point using Bioplex manager software. RLU/mL for each dilution of test samples were obtained by interpolation of the blank-subtracted MFI values at each specific dilution to a 5PLparameter logistic curve. Only values falling in the linear portion of the standard curve fitted using a 5PL-parameter were considered for the analysis. Interpolation of the standard curves allowed the blank-subtracted MFI values for samples to be converted to relative Luminex units (RLU/mL) for each specific dilution (i.e., by multiplying RLU/mL as interpolated from the standard curve by the specific dilution tested). To calculate the RLU/mL for each sample, the median RLU/mL of a minimum of three dilutions falling within the interpolation range for each sample was taken.

### 2.3. Assay Specificity

To determine the specificity of the multiplex assay, Privigen IVIG was incubated with each of the antigens prior to performing the assay to quantify homologous and heterologous inhibition. A total of 50 μL Privigen IVIG at 1:20,000 dilution (dilution in the linear range of 5PL curve) was incubated with 50 μL of each protein antigen and non-biotinylated GAC inhibitors, in serial 3-fold dilution from 30 μg/mL to 0.005 μg/mL alongside a well containing IVIG but no antigen inhibitor. Plates were incubated for 1 h at room temperature shaking at 750 rpm prior to adding 4-plex beads and proceeding with the standard assay described above. Percentage of inhibition, defined as = ((MFI Control − MFI Inhibited sample)/MFI control) × 100, for each concentration of inhibitor was calculated. To calculate heterologous inhibition, the % of inhibition, defined as = ((MFI Control − MFI Inhibited sample)/MFI control) × 100. MFI values used for calculation of percentage inhibition were the mean of two replicates. The concentration of each inhibitor antigen was determined, which achieved ≥80% homologous inhibition, and was then used to calculate the percentage of the heterologous inhibition of the remaining three antigens, with <20% heterologous inhibition defined as acceptable.

### 2.4. Limits of Standard Curve Accuracy and Lower Limits of Quantification

To determine upper and lower limits of standard curve accuracy for each antigen, duplicate IVIG standard curves from twelve independent assays were assessed. The MFI values from each experiment were used to fit a 5PL-parameter regression formula. Values falling outside the linear portion of the curve were ascribed the respective limit of quantification value for each individual experiment. The residual error (RE%) was calculated against the nominal concentration of the standard curve at each dilution of the IVIG standard ((calculated value − nominal value)/nominal value × 100), alongside 90% confidence intervals. Lower and upper limits of standard curve accuracy (LLSCA, ULSCA) were set at the nominal value of the IVIG standard at the lowest and the highest value, respectively, at which the predicted RE% with 90% confidence fell within an acceptable range of +/− 25%. Lower limit of quantification of the assay was set at the lower limit of standard curve accuracy multiplied per 100 (the lowest sera dilution tested in the assay).

### 2.5. Precision

In order to determine the precision of assay on different days and with different operators, a single pooled sample of human serum was tested independently with twenty-four repeats, by two operators on three different days. Each individual preparation of the sample was tested at three dilutions (1:3000, 1:9000 and 1:27,000) in the standard assay as described above, and antibody titres (expressed as RLU/mL) for each antigen were calculated from the median of values obtained from the three dilutions. Log_10_ transformed median RLU/mL for each of 144 separate tests were incorporated into a random-effects model alongside day and operator covariables, allowing calculation of the intermediate precision, repeatability and variance attributed to each factor via residual maximum likelihood method using MiniTab software (Minitab, LLC). Coefficients of variance for reliability and intermediate precision were then calculated from the log-transformed variance components and reverted to original RLU/mL units with the equation CV = √($e^{\left(\mathrm{sLn}\right)2}$ − 1), where sLn is calculated as standard deviation of log-transformed variance components multiplied by Ln (10).

### 2.6. Linearity

Assay linearity was determined from the pooled human serum, by performing independent dilutions (neat and 2-fold apart to 1:128) prior to testing in the standard assay. IgG antibody concentrations to each antigen were calculated from each dilution at three independent dilutions. Correlation between observed log_10_ transformed antibody titres and the log_10_ nominal antibody titre (represented by geometric mean of 144 separate measurements) divided by the dilution factor, was determined. 

### 2.7. Determination of Total Antigen Specific IgG in Individual Human Sera

Three separate pools (pool 1, 2 and 3, respectively) of anonymised sera, pooled from at least 30 (range 30–81) individual samples from children aged 24–59 months in The Gambia were obtained from a study performed for assessing immunological responses to live attenuated influenza vaccine [15]. Use of archived samples for assessing serological responses to Strep A was approved by the joint Gambia Government/Medical Research Council Unit, the Gambia Ethics Committee and the London School of Hygiene and Tropical Medicine Research Ethics Committee (ref 19163). Three different neat IVIG formulations Privigen, Octanorm and Intragram (CSL Bioplasma) were also tested together with IgG depleted human serum (Innovative Research, IIGGDS), which was included in the assay as a negative control. All samples were tested in both monoplex (where a single antigen-coupled microsphere region was incubated with the test samples) and multiplex (where all four antigen-coupled microsphere regions were incubated simultaneously with test samples). Correlation between monoplex and multiplex IgG titres with log_10_ transformed RLU/mL was performed in Minitab software through fitting a linear regression model.

## 3. Results

### 3.1. Setup of Standard Curve

**Figure A1:**
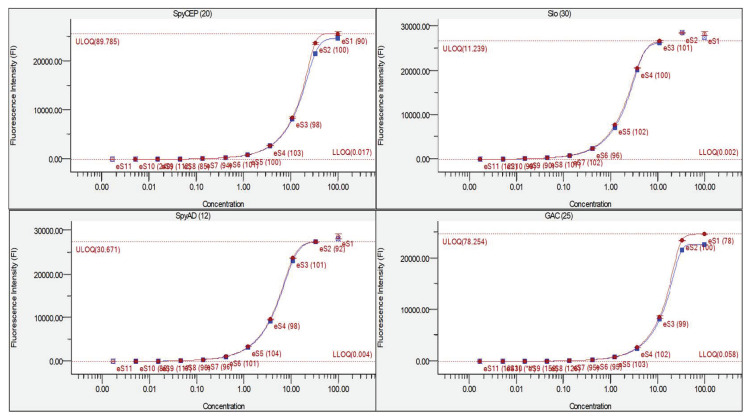
Comparison of Privigen (red) with Octanorm (blue) standard curves fitted with 5-PL regression model. IVIG serially diluted 3-fold from 1:1000 dilution (nominal concentration 100 RLU/mL) to 1: 1:6,561,000. Initial assay conditions were 30 min of primary incubation, 30 min of secondary incubation with 1:70 dilution of secondary antibody.

**Figure A2:**
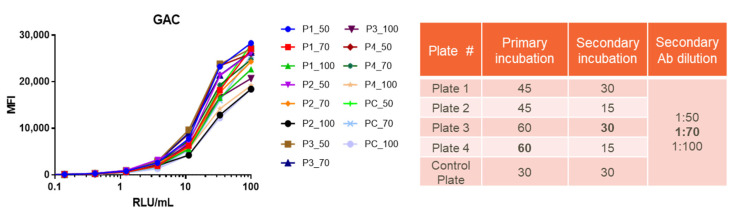
Standard curve optimisation (GAC only shown) with Privigen IVIG serially diluted 3-fold from 1:1000 dilution (nominal concentration 100 RLU/mL) to 1: 1:6,561,000.

In order to create a fully quantitative assay it was essential to establish and fully characterise the performance of a working standard, whereby robust assay parameters were established. This allows comparison and quality control between each plate, over time and between laboratories in order to standardise measurements obtained from the assay. For multiplex assays the standard must contain antibodies to all included antigens in sufficient and comparable concentrations. This may be achieved via pooling different concentrations of monovalent standard or via the identification of the standard material with well-balanced concentrations of antibodies to all antigens. Therefore, two formulations of pooled human immunoglobulin (IVIG), Privigen and Octanorm, both commercially available, were assessed as working standards. For both formulations we demonstrated similar curve appearances (Figure A1) and IgG measurement of test samples interpolated from the standard curves (not shown). Privigen IVIG was selected for the working standard due to greater commercial availability. The starting dilution of 1:1000 for the working standard was selected to avoid the prozone effect and obtain a well-fitted 5PL sigmoidal curve with all four antigens, as determined in preliminary setup experiments, in which the starting dilution of the standard was evaluated from 1:100 dilution, 23 dilution points 2-fold apart (data not shown). The final working standard was determined with duplicate 10-point 3-fold serial dilutions of Privigen IVIG, commencing at 1:1000, which was assigned an arbitrary value of 100 relative Luminex units/mL (RLU/mL) (Figure A2). The optimal assay conditions were determined with 60 min primary incubation and 30 min incubation of the secondary-PE conjugated antibody at 1:70 dilution (Figure A2).

### 3.2. Determination of Standard Curve Accuracy and Limit of Quantification of the Assay

**Figure A3:**
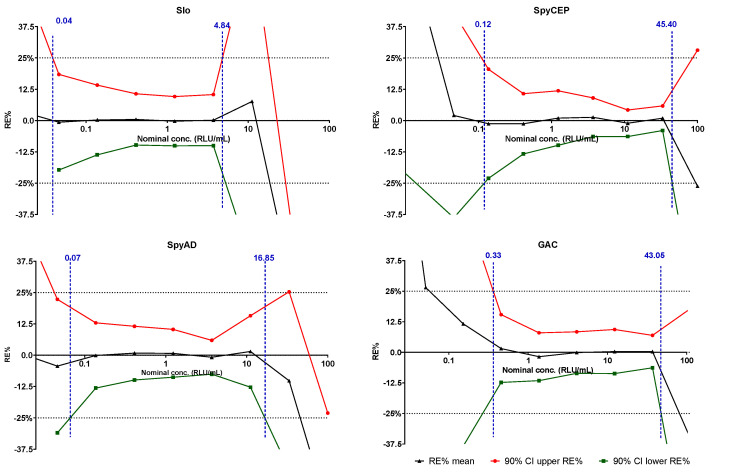
**Accuracy assessment.** % relative error (black line) with 90% upper(red) and lower (green line) confidence intervals, against nominal concentration of standard curve from 12 independently performed assays. From the plotted values the upper and lower limits of standard curve accuracy could be interpolated from with a conservative threshold where there is a 90% probability that the relative error is <25%.

To determine standard curve accuracy, duplicate standard curves from twelve independent assays were used to assess the relative error and 90% confidence intervals of the standard concentration at different nominal values (in RLU/mL) of the standard curve (Table 1, Figure A3). The lower limit of quantification (LLOQ) was calculated for each antigen by multiplication of the lower limit of the standard curve accuracy (LLSCA) by the lowest dilution factor of any test sample (1:100) by the LLOQ for each antigen (Table 1). 

### 3.3. Specificity

**Figure 1:**
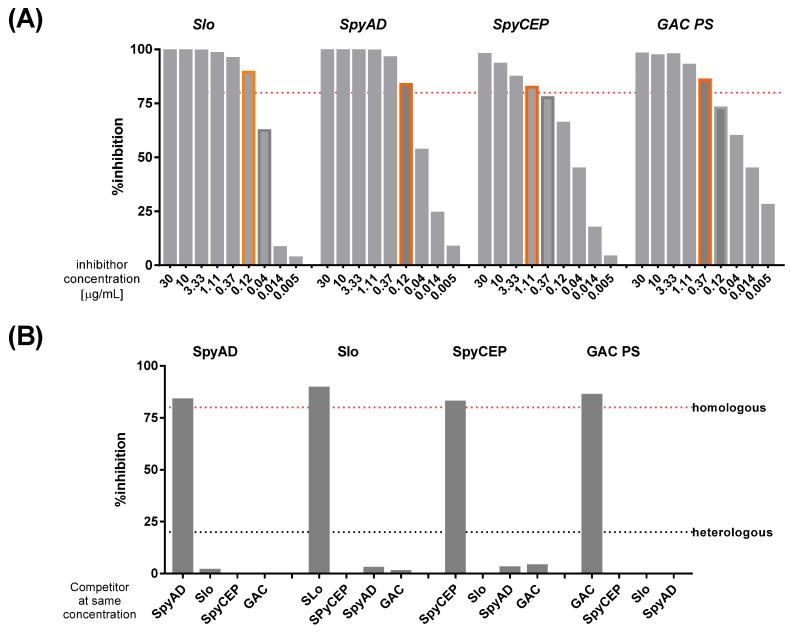
**Assay specificity.** (**A**) Percentage inhibition of MFI for each antigen after incubation with its homologous inhibitor at varying concentrations (in orange highlighted the lowest concentration of homologous competitor leading to >80% inhibition). (**B**) Percentage inhibition of homologous and heterologous 4-plex antigens at concentration of inhibitor which led to >80% inhibition of its homologous antigen in 1A. The figure depicts a single specificity experiment with a single batch of purified protein and group A Carbohydrate antigens. Percentage inhibition calculated from the average of two repeats of each antigen at each concentration.

To determine the specificity of the assay, Privigen IVIG was incubated with different concentrations of homologous competitor antigens (Slo, SpyAD, SpyCEP and GAC) prior to testing with the standard assay. The percentage of inhibition at each concentration of the competitor was calculated in comparison with an uninhibited sample under the same conditions for each antigen-coupled microsphere region (Figure 1A). The lowest concentration of the homologous competitor able to inhibit the signal by at least 80% was selected for further experiments, in which standards were incubated with homologous competitors and heterologous competitors at the same concentration prior to testing with the standard assay. High specificity was established for measurement of IgG to all four antigens, as percentage inhibition was >80% after incubation with homologous competitors and <20% in presence of each of the heterologous competitors (Figure 1B).

### 3.4. Precision

**Figure 2:**
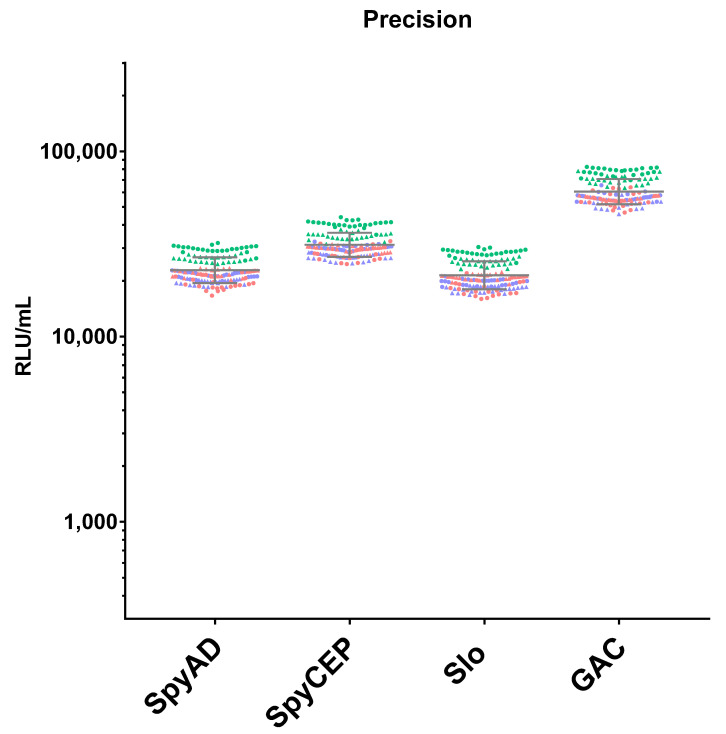
**Assay precision**. A total of 144 repeated measurements of IgG titres from a single independently handled sample, by two operators on three different days. Single repeats of each operator are represented by circle symbols (for operator 1) and triangles (for operator 2), repeats on different days are shown in orange for day 1, green for day 2 and blue for day 3. Geometric means and geometric standard deviations from 144 repeats are represented by the grey lines for each of the antigens.

Analysis of the precision of the assay was performed on 144 independently handled repeat tests of a single sample, by two operators on three days (24 independent repeats per day by each of the two operators), and the results are shown in Figure 2. Analysis of the variance components via a random-effects model incorporating day and operator covariables (Table 2), showed that there was no significant contribution to the variance component made by day, operator nor the interaction between day and operator for the measurement of IgG to any of the antigens (*p* at least 0.11 for the significance of variance components for each covariable).

### 3.5. Linearity

**Figure 3:**
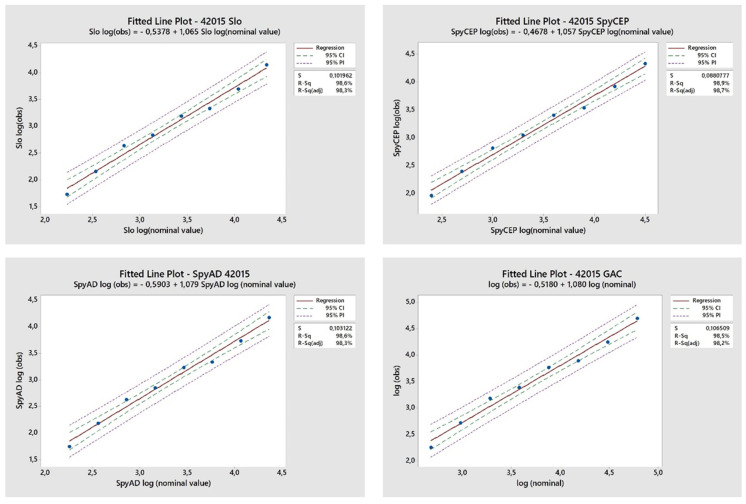
**Linearity assessment**. A single sample was independently diluted from neat to 1:128 and tested in the assay as separate samples. Correlation between observed IgG titres (log_10_ RLU/mL) and the nominal antibody titre was determined. Nominal antibody titre was defined as the mean IgG titre (log_10_ RLU/mL) of 144 separate tests performed on that sample during precision analysis, divided by the dilution factor.

Linearity was assessed by testing samples independently diluted (eight dilution points, 2-fold apart, from neat to 1:128 diluted sample) prior to performing the standard assay considering each dilution a test sample. A high correlation between observed IgG titres and nominal IgG titres at each specific dilutions was observed for all four antigens across a range of dilutions from neat to 1:128, treated independently in the assay (Figure 3).

### 3.6. Quantification of IgG Titres in Human Serum

**Figure 4:**
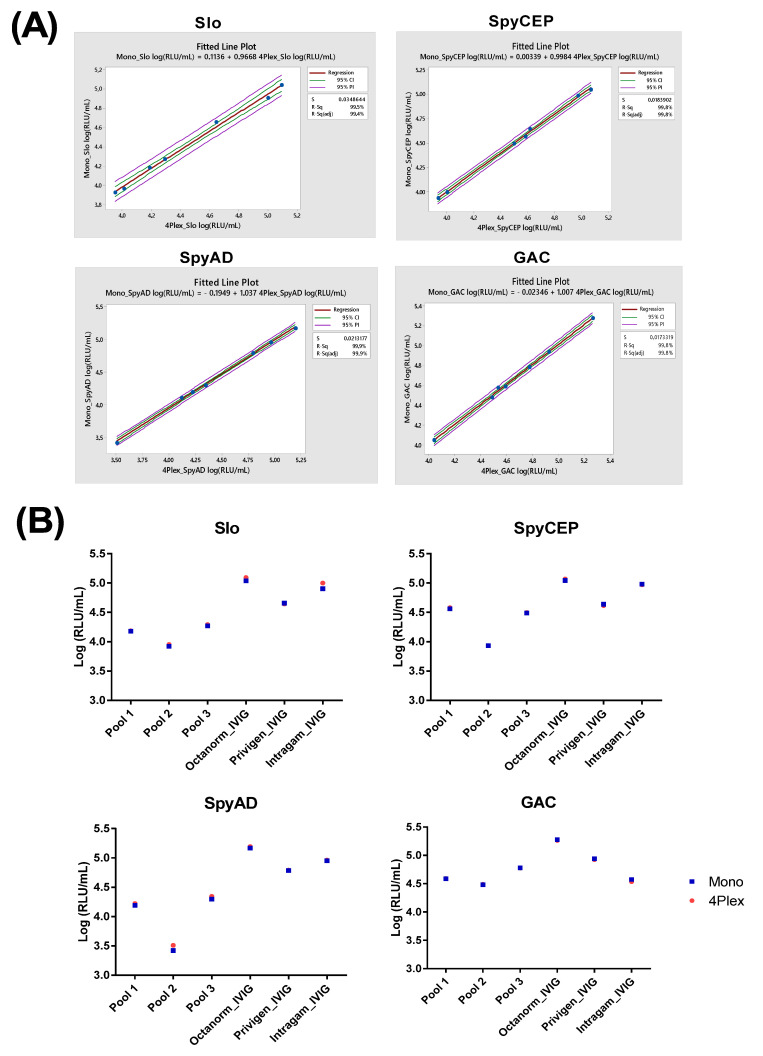
**Assessment of IgG concentrations measured in monoplex and multiplex assay for four vaccine antigens.** Figures demonstrate a single experiment comparing monoplex and multiplex to measure IgG. RLU/mL values were determined from the median RLU/mL from four dilutions of each sample in both monoplex and multiplex. (**A**) RLU/mL values were log_10_ transformed prior to correlation analysis with linear regression. (**B**) Direct comparison of IgG concentrations measured in monoplex and multiplex assay for 4 vaccine antigens testing three different pools of human sera (pool 1, pool 2 and pool 3, respectively) and three different pools of commercial IVIG (Octanorm, Privigen and Intragam, respectively).

Finally, three different human-pooled sera and IVIG were tested as samples to compare the performance of the monoplex assay to multiplex assay, demonstrating high correlation and linearity between values obtained in monoplex and multiplex (Figure 4A), as well as the ability to appropriately determine antibodies with a broad range of concentrations (Figure 4B).

**Figure A4:**
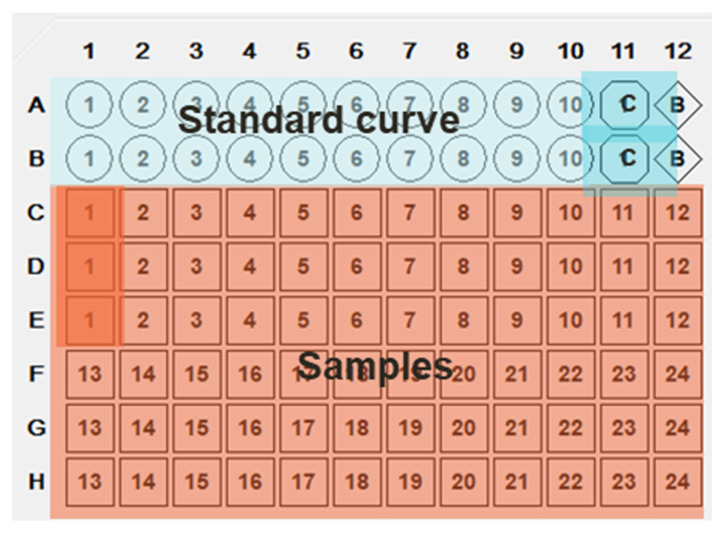
Suggested 96 well plate layout for testing of clinical samples in future human vaccine trials and seroepidemiological studies. C = positive control, B = blanks.

Based on our results, a potential plate layout for clinical testing, balanced for throughput and assay performance, will consist of a 10-point standard curve prepared with IVIG run in duplicate, plus two control wells (control sera at specific dilution, whose RLU/mL needs to be in a specific range to validate the entire plate) and two blank wells (Figure A4). Up to 24 samples will be tested in 3-fold serial dilution with results represented by the median RLU/mL obtained between three dilution points for each sample, provided that all fall within the limits of standard curve accuracy and quantification.

## 4. Discussion

With the pressing needs for the development of an effective and safe vaccine to reduce the burden of Strep A mediated disease, substantial efforts are being made by organisations including the World Health Organisation, the Strep A Vaccine Global Consortium and the Australian Strep A Vaccine Initiative to accelerate development of a Strep A vaccine [16,17]. Recommendations from the WHO roadmap towards a Strep A vaccine and from the alliance of global stakeholders clearly highlight the need for standardised immunoassays to measure antibody responses to Strep A vaccination [4,18]. It is also widely recognised that progress towards a Strep A vaccine is limited by poor understanding of the naturally occurring immunity and pathological immunity leading to RHD [19]. It is, therefore, imperative that transferrable assays are available to organisations conducting vaccine trials and the wider research community, to measure vaccine responses from clinical trials, as well as naturally occurring antibodies from both seroepidemiological studies and from longitudinal studies of natural immunity. Luminex platforms have proven a popular choice given their relatively high throughput and transferability to LMIC research institutions, where research will need to take place to address the needs of LMIC populations who experience by far the greatest burden of Strep A-related disease. [1,2,20].

We describe the in-depth characterisation of a 4-plex assay to quantify IgG in human sera to four vaccine antigens using the Luminex platform. We have firstly demonstrated that the assay is highly specific with limited nonspecific reactivity between antigens, comfortably meeting prespecified criteria for specificity. Furthermore, the assay was precise, repeatable and performs linearly across a large concentration range. The dynamic range of the standard curve is high; thus, the assay can accurately quantify IgG titres across a large range of concentrations at a given test sample dilution. The limits of quantification have been established with low limits of quantification for each antigen, comparable with other published assays using the Luminex platform to measure IgG to Strep A antigens coming from different settings [11]. Furthermore, the detailed standard curve characterisation can be used to standardise the measurement of IgG to the four vaccine antigens between plates, over time and to compare assays performed in different laboratories, thereby facilitating the transfer of the assay, including to laboratories in LMIC settings. The assay described will be suitable for measuring serological responses in both vaccine trials and studies of natural immunity [4].

This manuscript describes the technical characterisation of an assay to the standards required for vaccine studies to measure IgG in human serum to the antigens contained in a candidate vaccine in development at GVGH. Although this manuscript is limited by a lack of cohorts from which the assay can be validated and evaluated, we describe a well performing assay that can now be employed to reliably and accurately measure IgG responses to vaccination in humans and in well described clinical cohorts. The measurement of IgG to Strep A antigens via the Luminex platform has increasingly demonstrated promise for vaccine trials, observational cohort studies alike [10,11], and for the characterisation of humoral immune responses from clinical Strep A syndromes, as well as acute rheumatic fever [21]. Although it is well recognised that a serological correlate of protection is not established, which limits the interpretation of immunological outcomes in potential vaccine trials [8], Luminex assay here described may pave the way for powerful and reliable tools in the identification of serological correlates of protection and specific rheumatogenic signatures. High throughput serological techniques building from this method, and others will play an important role in vaccine trials and longitudinal studies of natural and pathological immunity to Strep A.

## Figures and Tables

**Table 1:** **Standard curve performance characteristics.** Limits of standard curve accuracy and lower limit of quantification to measure IgG to four vaccine antigens were calculated from 12 independent assays. LLSCA = Lower limit of standard curve accuracy, ULSCA = Upper limit of standard curve accuracy. LLOQ = Lower limit of quantification.

Antigen	LLSCA (RLU/mL)	ULSCA (RLU/mL)	LLOQ (RLU/mL)
Slo	0.04	4.84	12
SpyCEP	0.12	45.4	36
SpyAD	0.07	16.85	21
GAC	0.33	43.05	99

**Table 2:** **Repeatability and intermediate precision calculation.** No significant contribution to the variance component was observed by day, operator nor the interaction between day and operator.

Antigen	Repeatability CV (%)	Intermediate Precision CV (%)
Slo	5.2	20.8
SpyCEP	4.8	17.7
SpyAD	5.2	18.9
GAC	5.7	18.6

## Data Availability

Not applicable.

## Appendix A

**Table A1:** Antigen coupling concentration and MagPlex bead region used for each antigen.

Antigen	Name	Coupling Concentration (μg per 1.25 × 10^6^ Million Beads)	MagPlex Bead Region
**Slo**	Streptolysin O	10	30
**SpyCEP**	*S. pyogenes* cell envelope protein	10	20
**SpyAD**	*S. pyogenes* adhesion and division protein	20	12
**GAC**	Group A carbohydrate	10 (20 μg of streptavidin)	25
